# Hybrid framework for image forgery detection and robustness against adversarial attacks using vision transformer and SVM

**DOI:** 10.1038/s41598-025-25436-z

**Published:** 2025-11-18

**Authors:** Mohamed Abdelmaksoud, Basheer Youssef, Khaled Wassif, Reda A. El-Khoribi

**Affiliations:** Department of Computer Science, Faculty of Computers and Artificial Intelligence, Cairo University, Cairo, Egypt

**Keywords:** Forgery detection, Vision transformer, Support vector machine (SVM), Adversarial attack, Adversarial training, Engineering, Mathematics and computing

## Abstract

People routinely capture photos and videos to document their daily experiences, with such visual media frequently regarded as reliable sources of evidence. The proliferation of social networking platforms, digital photography technologies, and image manipulation applications have introduced emerging concerns that demand investigation by academics, industry executives, and cybersecurity experts. These concerns specifically relate to identifying and mitigating fraudulent visual content across online platforms. The deliberate alteration of photographs and videos has become progressively prevalent, potentially resulting in severe emotional, bodily, and societal damage to affected persons. This research introduces a combined Deep Learning approach utilizing a pre-trained Vision Transformer (ViT) for feature extraction alongside Support Vector Machine (SVM) for dual-category image classification, differentiating authentic from manipulated photographs (Copy-move & Splicing). Additionally, we implemented adversarial training techniques to enhance model robustness against adversarial attacks. The introduced approach underwent comprehensive evaluation across multiple benchmarks, including CASIA v1.0, CASIA v2.0, MICC-F220, MICC-F2000, and MICC-F600. The methodology exhibits considerable potential regarding forgery detection performance following extensive validation. The proposed framework demonstrated competitive performance and improved robustness against image manipulations compared to existing methods in manipulation detection tasks.

## Introduction

Growing concerns exist about the accuracy and genuineness of visual content due to the common use of digital imaging technology and photo editing software^1,2^. Today, individuals and organizations can easily create altered photographs and videos that look identical to authentic ones^3,4^. This increase in creating and sharing manipulated images and videos causes serious problems in areas like social media, forensics, journalism, and law enforcement, where visual accuracy is essential^5^. The availability of photo and video editing tools makes it harder to distinguish between real and fake images. The widespread creation and modification of images and videos can lead to various harmful outcomes, including spreading false information, reducing public trust.

Digital image forging is when you add strange patterns to original images that change the way the image properties seem and how the image features are spread out. Digital image forgeries appear in many forms, such as copy-move forgery and other morphological applications like retouching, splicing, morphing, and resampling. Digital photograph manipulation falls into two categories: active and passive approaches. Active methods necessitate prior information regarding the image during authentication procedures. The embedded data within photographs serves to establish image origin or detect alterations, encompassing two approaches: digital signatures and digital watermarking. Conversely, passive detection methods function independently of pre-inserted information, examining statistical, compositional, or artifact-related anomalies. Texture analysis within photographs helps detect irregularities, indicating potential manipulation techniques (image splicing and copy-move operations). This investigation examines two prevalent photograph manipulation methods: splicing and copy-move techniques.

### Copy-move:

Copy-move manipulation consists of duplicating single or multiple regions from an image and relocating them to alternative positions inside the identical image^6^. Such manipulation serves to conceal details or replicate entities or individuals, thereby substantially modifying the interpretation and substance of the original image.

### Splicing:

Splicing forgery differs from copy-move forgery in that the copied objects or regions come from different images^7^. This type of forgery can create false scenarios or hide specific content.

**Fig. 1:**
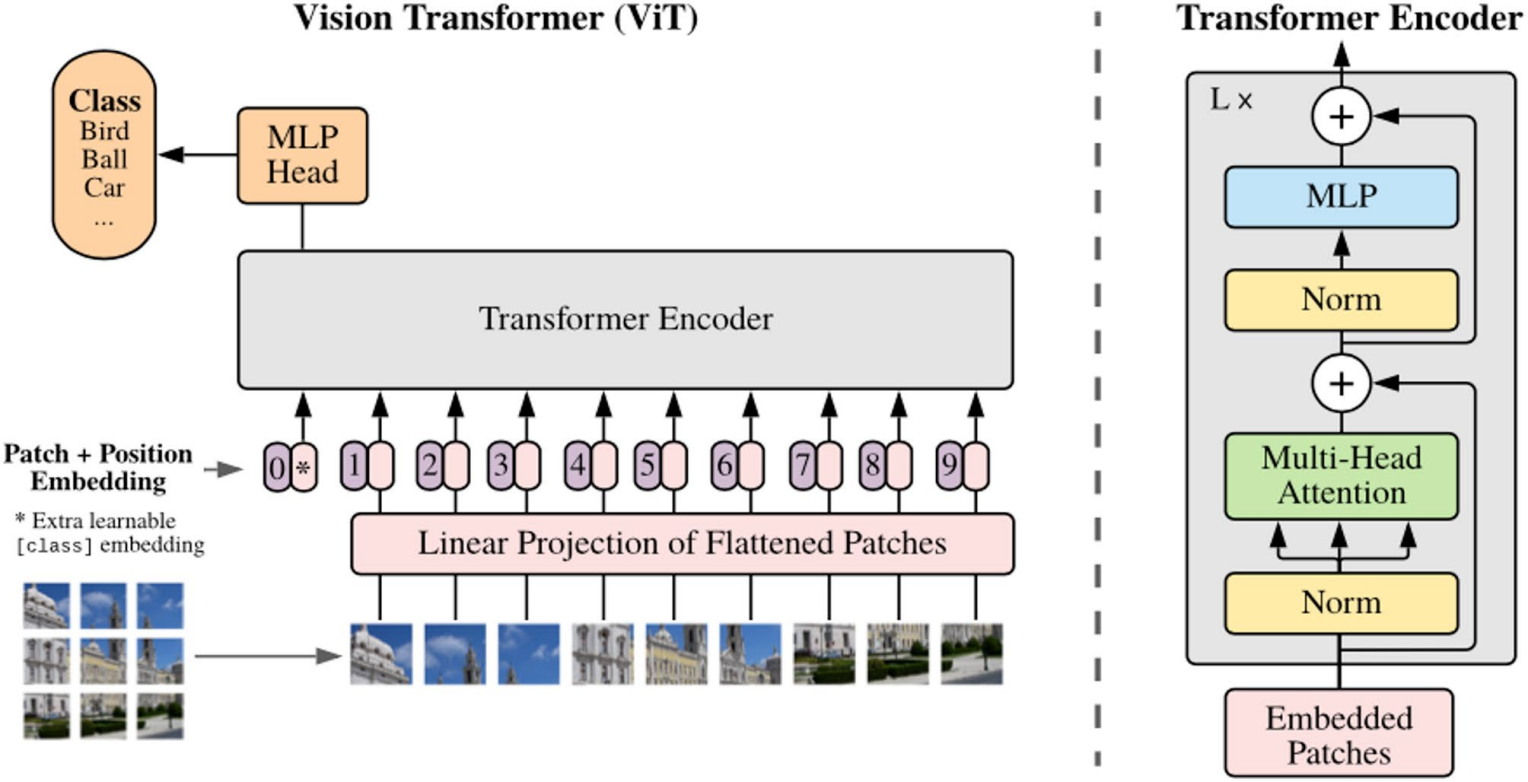
Vision transformer (ViTs) architecture^8^.

The Vision Transformer (ViT) modifies the Transformer framework—initially developed for natural language processing —for computer vision tasks through segmenting input images into uniform patches, subsequently projecting them linearly as patch embeddings. These embeddings include learnable positional encodings to retain spatial information prior to processing by standard Transformer encoding blocks, including multi-head self-attention (MHSA) and multilayer perceptrons (MLPs). Vision Transformers (ViT) have revolutionized computer vision by their capacity to capture global relationships and long-range spatial dependencies present within images, in contrast to convolutional neural networks (CNNs). VisionTransformers (ViTs) have shown excellent performance over a range of tasks such as object detection, image classification, image segmentation, etc. However, although ViT shows strong performance during training on large-scale repositories (i.e. ImageNet-21k or JFT-300 M), its dependency on large amounts of data and heavy computing makes it impractical in many situations. The architecture of the Vision Transformers (ViTs) is given in Fig. 1.

Adversarial attacks are ways of creating adversarial samples. An adversarial sample is an intentionally altered machine learning input that was created to generate a misclassification while still seeming valid to a human viewer. In order to trick deep learning networks, adversarial attacks include adding crafted perturbations to input images. Due to their dependence on self-attention mechanisms and patch-based processing, Vision Transformers (ViTs), although resilient in some situations, are nonetheless susceptible to adversarial attacks.

**Fig. 2:**
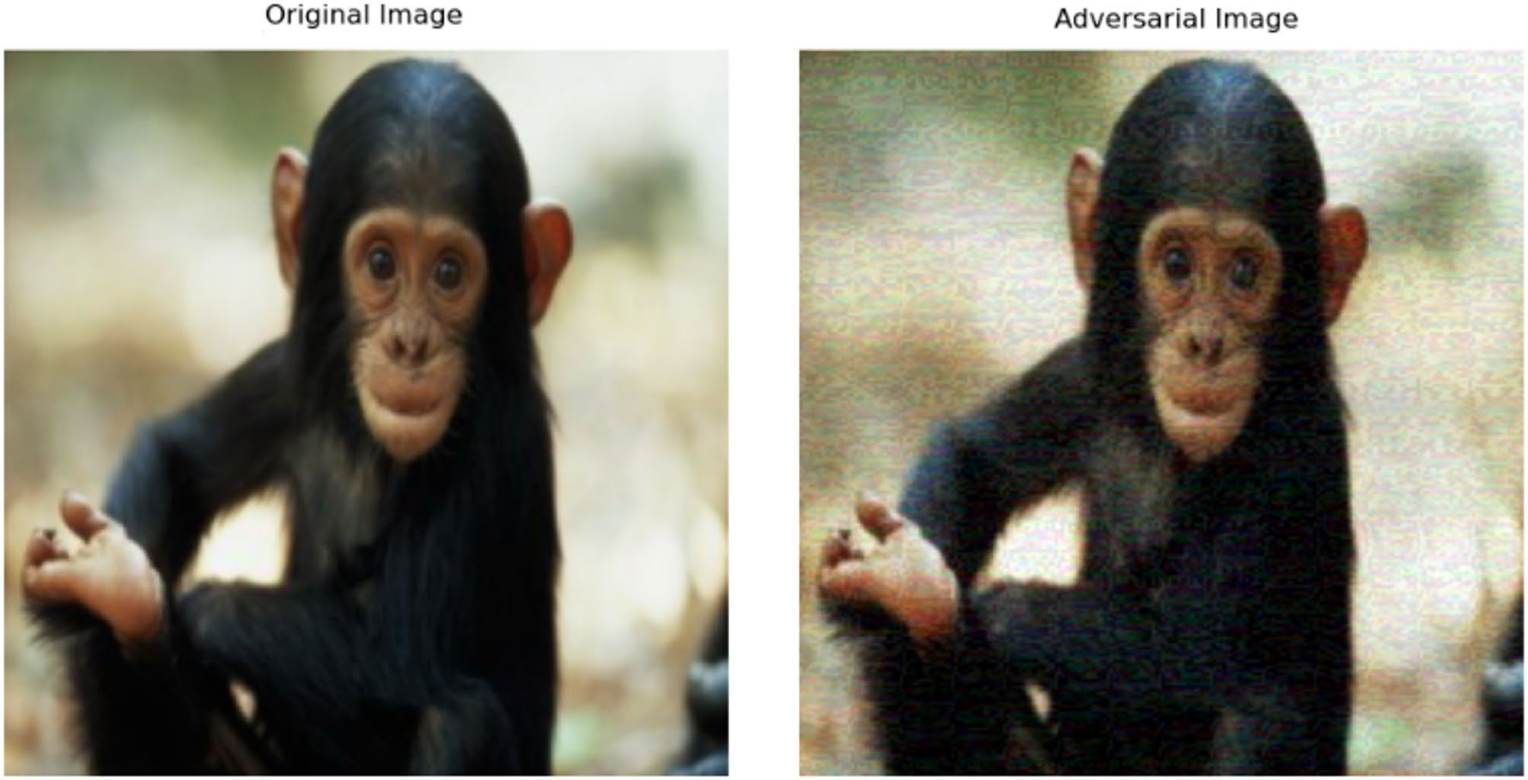
Example of patch fool attack.

The Patch-Fool attack^9^ is an adversarial attack designed to exploit ViTs’ patch-based processing. The Patch-Fool attack is a targeted adversarial attack on Vision Transformers (ViTs) that strategically perturbs only a few critical image patches— identified via attention or gradient importance—to maximally disrupt the model’s self-attention mechanism, achieving high deception rates with sparse, localized noise while remaining computationally efficient, as shown in Fig. 2. To defend against it, we will use adversarial training. Adversarial training enhances network resilience through incorporating adversarial modified samples into training collections, compelling networks to produce accurate classifications despite hostile input modifications.

The contribution of this paper is to propose a novel deep learning method for image forgery detection and Robustness against adversarial attacks using ViT and SVM. The contributions in the following steps:

- **Dataset augmentation**: To the best of our knowledge, we are the first to merge and evaluate across five standard forensic datasets (CASIA v1.0, CASIA v2.0, MICC-F220, MICC-F2000, MICC-F600), creating a unified benchmark for broader generalizability.
- **Adversarial robustness**: Unlike many existing works, we explicitly evaluate and enhance robustness against adversarial attacks, including Patch-Fool perturbations, which specifically target transformer-based architectures.
- **Hybrid ViT-SVM framework**: Our approach freezes the pre-trained Vision Transformer (ViT) for feature extraction and integrates a Support Vector Machine (SVM) for classification. This differs from prior end-to-end transformer fine-tuning approaches and provides both computational efficiency and interpretability.
- **Transfer learning efficiency**: We leverage transfer learning from large-scale pretraining, allowing ViT to capture rich semantic and spatial cues that significantly aid forgery detection.

The remainder of this paper is structured according to the following: Section II clarifies related works in these fields. Subsequently, the proposed method in section III. Section IV includes the result. Section V includes discussion. Finally, Section VI presents the conclusion and future work.

## Related work

In this section we divide the related work into four category.

- Convolutional Neural Networks Based.
- Convolutional Neural Networks with Transfer learning.
- Vision Transformers Based.
- Hybrid Approaches.

### Convolutional neural networks based

Deep neural networks are designed to perform both feature extraction and classification within the same system. When properly initialized and trained with appropriate parameters, deep neural networks (DNNs) can identify important features and accurately classify images. In this approach, features are extracted directly from the deep network and are called deep learning-based features. Sometimes, manually designed features are also input into the DNN to reduce training time and improve accuracy. Deep learning networks can overcome the limitations of traditional or manually created features by automatically learning numerous features^10^. Deep learning-based image detection methods train models using many authentic and manipulated images to detect tampering^11^. Through well-designed neural network architectures, deep learning networks have shown effectiveness in finding complex hidden patterns in data that can separate forged areas from authentic parts of an image.

Verma et al.^12^ present a forgery identification approach integrating convolutional neural networks (CNNs) alongside error level analysis (ELA). ELA represents a processing technique for detecting anomalies and potential alterations within photographs. The methodology implements the ELA technique prior to processing photographs through CNN architectures. Their technique obtains 94% precision during evaluation using the benchmark CASIAv2 dataset, comprising 7,491 genuine photographs and 5,123 manipulated photographs. The authors in^13^ construct a framework employing a convolutional neural network (CNN) utilizing YOLO weights and ResNet50v2 structure for analyzing input photographs. Their approach undergoes testing across CASIA v1 and CASIA v2 repositories, obtaining 85% precision. The architecture presented in^26^ incorporates two integrated phases: feature extraction and manipulation identification. Within the feature extraction phase, multiple features were carefully extracted, encompassing SURF, DWT, LVP, PCA, HoG, and MBFDF. The extracted features functioned as CNN inputs, utilizing weight optimization through the Sealion Customized Firefly algorithm (SCFF). In^24^, the authors proposed a neural network method for the detection of photograph manipulations. The framework is trained using the difference between original and recompressed image versions to give an efficient and easy-to-understand forgery identification scheme. In^28^, the authors introduced an efficient and fast CNN model designed for copy-move manipulation detection. Although the framework was conceptually simple, it identified significant levels of precision in identification, which demonstrates its ability to detect altered images.

### Convolutional neural networks with transfer learning

Transfer learning is a computational method that uses previously trained architectures on initial problems as starting points for related downstream tasks. This approach is particularly useful when the secondary applications have limited samples or when applications have similarities. By using patterns learned from previous applications, architectures can be modulated efficiently toward new goals, generating faster training and higher accuracy. Neural network models require large numbers of training samples. Nevertheless, accessible repositories for photograph manipulation detection include comparatively limited photographs. Such constraints encourage investigators to implement transfer learning for manipulation detection, enabling weight utilization from current trained architectures rather than training the neural network architectures from scratch^17^.

Two deep learning methods for detecting copy-move forgeries were proposed in^18^. Model 1 was based on a custom-designed architecture, and Model 2 was based on VGG-16 with transfer learning. To obtain better generalization, the images from eight different publicly available datasets were used. The proposed architecture solved the problem of the generalization of forgery detection by training on one dataset and testing on every other dataset. In^19^, researchers proposed a deep learning approach for image splicing detection which consisted of three steps: (1) preprocessing, which included RGB conversion and the resizing of images; (2) feature extraction with a pretrained AlexNet model; and (3) classification in which classifier called Canonical Correlation Analysis (CCA) was trained on extracted features for binary classification (real/fake). In^20^, a blind image splicing detection method based on a deep convolutional residual network architecture is proposed. ImageNet weights are used for initialization, and the experiments were done on the ResNet-50 architecture for the extraction of initial image features, while fully connected layers are used for the classification task.

While CNN-based approaches have demonstrated satisfactory performance, their constraints include being vulnerable to post-processing modifications (e.g., compression artifacts, noise interference, and resolution changes) and diminished stability when confronting novel manipulation techniques. These shortcomings restrict their adaptability across varied forgery contexts and susceptibility to adversarial attacks.

### Vision Transformers based

Vision Transformers possess the capability to examine entire images for identifying forgeries across different scales, from minor to extensive manipulations. Through robust feature extraction and self-attention mechanisms, ViTs demonstrate enhanced performance in concentrating on critical regions affected by image tampering. Unlike CNNs that depend on localized filtering approaches, Vision Transformers can accommodate various forgery types, including those produced through artificial intelligence-based manipulations. Han et al.^29^ present a comprehensive overview of advancements in ViT architecture, covering essential aspects such as training methodologies, practical applications, and architectural design principles, offering valuable insights into the evolution of Vision Transformer technology. This review serves as an excellent resource for understanding the contemporary landscape of ViT research.

Chen et al.^30^ introduced an innovative CrossViT architecture, representing a variant of vision transformer (ViT) that explores multi-scale feature representations within transformer frameworks for image classification tasks. This dual-branch transformer employs attention mechanisms to merge images through processing patches of varying dimensions. The authors proposed a cross-attention-driven token fusion component to enhance model performance, leading to significant computational time savings compared to quadratic complexity operations.

Pawar et al.^31^ employ a Vision Transformer (ViT) architecture for binary classification to distinguish between authentic and manipulated images. Additionally, they utilize a pre-trained Segment Anything Model (SAM) that undergoes fine-tuning with custom datasets to identify and localize forged regions within images. Comprehensive evaluation across multiple benchmark datasets including CASIA v1.0, CASIA v2.0, MICC-F2000, MICC-F600, and Columbia demonstrates the method’s effectiveness in both classification accuracy and localization precision, making it a valuable tool for multimedia forensics applications.

Despite their robust performance, ViTs encounter constraints, including reliance on extensive training datasets, elevated computational requirements, and vulnerability to adversarial attacks. Furthermore, numerous ViT-based methodologies concentrate solely on classification tasks, without considering robustness factors or computational efficiency necessary for real-time forensic applications.

### Hybrid approaches

Hybrid approaches combine more than one method like, machine learning techniques with deep learning, for improved forgery detection. Siddiqui et al.^15^ incorporate the DiffPIR diffusion model to improve image clarity, particularly enhancing facial characteristics to facilitate superior feature extraction. Subsequently, a CNN architecture based on DenseNet121 is utilized to differentiate between authentic and synthetic frames, including AI-generated content, by concentrating on facial regions. Evaluation on established benchmark datasets including Celeb-DF and DFD reveals that the approach surpasses current state-of-the-art techniques, attaining classification accuracy exceeding 99.91%.

Akram et al.^16^ introduced a hybrid methodology that identifies copy-move forgery within digital images through the integration of FFT filtering techniques, SIFT, and ORB keypoint detection, combined with deep learning architectures, including MobileNetV2 and VGG16, enhanced by attention mechanisms. Keypoint correspondence is established using Euclidean distance measurements, while DBSCAN clustering algorithms pinpoint tampered areas, ensuring the approach maintains resilience against various image degradations such as blur effects, compression artifacts, luminance variations, and contrast modifications.

Siddiqui et al.^14^ introduced an innovative deepfake detection approach that combines DenseNet121 architecture with Vision Transformers to identify sophisticated facial manipulations in video content. The methodology incorporates a straightforward yet powerful voting-based inference system to address scenarios involving multiple facial regions within individual video frames. Performance evaluation demonstrates exceptional results, with F1-scores reaching 99.0% on the DeepForensics 1.0 dataset and 95.1% on CelebDF.

While hybrid approaches frequently attain superior accuracy through combining various techniques, their drawbacks encompass enhanced architectural complexity, elevated training expenses, and insufficient systematic evaluation of adversarial robustness. Additionally, numerous studies conduct assessments on restricted datasets without examining generalization performance across varied manipulation categories.

## Proposed method

### Introduction to the proposed method

The proposed method provides a novel solution for the detection of image manipulation by combining efficient data pre-processing techniques with the implementation of a bi-architecture classification system. It attempts to compensate for the lack of training samples and requires efficient feature extraction. The approach incorporates the power of modern transformer frameworks combined with traditional machine learning techniques to achieve better outcomes when classifying original versus modified photographs.

Th proposed methodology aims to contribute to the field of image forensics by:

1. Providing a robust framework that can handle diverse types of image manipulations.
2. Demonstrating the effectiveness of combining transformer-based feature extraction with traditional classifiers.
3. Establishing a comprehensive data preparation pipeline that enhances model generalization.
4. Offering a computationally efficient solution suitable for real-world applications.

### Overall framework architecture

The proposed methodology consists of two distinct but interconnected phases, as illustrated in the system architecture:

#### Phase 1 (Dataset processing and Preparation phase):

This phase focuses on enhancing the training dataset through data augmentation techniques and adversarial training methods. The primary objective is to create a comprehensive design domain dictionary that captures diverse variations and potential attack scenarios.

#### Phase 2 (Hybrid framework phase):

This phase implements the core classification system using a combination of Vision Transformer (ViT) for feature extraction and Support Vector Machine (SVM) for final classification. The hybrid approach capitalizes on the strengths of both deep learning and traditional machine learning techniques.

#### Phase 1: dataset processing and Preparation

The first phase of the proposed scheme focuses on building a large and augmented training dataset using data augmentation techniques. And this phase is still necessary to ensure that the final created classification scheme achieves good generalizationtoward the unseen samples and maintains robustness against different attack variants.

##### Data augmentation technique implementation

**Fig. 3:**
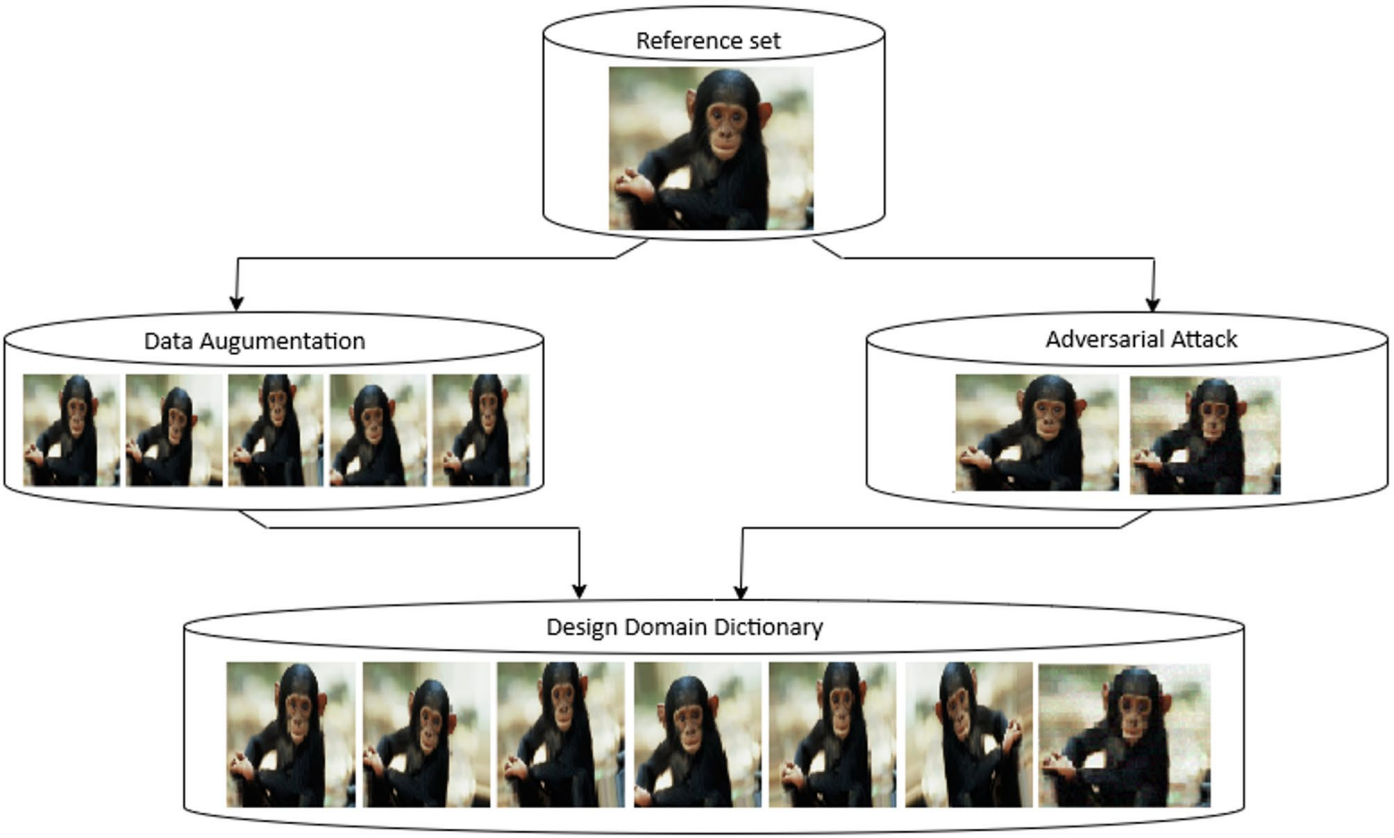
Dataset preparation phase: original images are expanded using data augmentation and adversarial samples (Patch-Fool), producing the design domain dictionary for training.

Data augmentation serves as the foundation for expanding the training dataset. As demonstrated in Fig. 3, this technique applies various transformations to existing images, effectively multiplying the dataset size while introducing controlled variations that improve model robustness.

**Geometric Transformations:** The following geometric transformations are systematically applied to each image in the original dataset:

- **Rotation**: Images are rotated at various angles (± 15°, ± 30°, ± 45°) to simulate different viewing perspectives and camera orientations.
- **Translation**: Horizontal and vertical shifts are applied (± 10%, ± 20% of image dimensions) to account for different cropping scenarios.
- **Scaling**: Images undergo scaling using coefficients between 0.8 and 1.2 for replicating various magnification settings and photographic distances.
- **Flipping**: Both horizontal and vertical flips are applied to increase directional diversity.
- **Shearing**: Shear transformations with angles up to ± 20° are applied to simulate perspective distortions.
- **Zooming**: Random zoom operations (0.9x to 1.1x) are performed to create scale variations.

**Augmentation Benefits:** The systematic application of these transformations provides several advantages:

- Increases the effective size of the training dataset by a factor of 6–8.
- Incorporates practical variations potentially encountered during actual applications.
- Enhances framework generalization through exposure to varied image representations.
- Minimizes overfitting through preventing network memorization of particular image alignments.

Six augmented samples were created from each original image through various transformations such as rotations, translations, scaling, flips (both horizontal and vertical), shearing, and zoom adjustments. This structured approach promoted dataset diversity and helped avoid overfitting.

##### Adversarial training implementation

The second component of the dataset processing phase involves adversarial training, which significantly enhances the model’s robustness against malicious attacks.

**Patch-Fool Attack Generation:** The adversarial training employs Patch-Fool attacks to generate adversarial examples:

- Small perturbations are added to create adversarial samples.
- These patches are designed to fool detection systems.
- The attack parameters are varied to create diverse adversarial scenarios.

**Adversarial Training Benefits**

- Forces the model to learn features that are robust to small perturbations.
- Improves resistance against adversarial attacks in real-world deployment.
- Enhances the model’s ability to focus on genuine forgery.
- Increases overall model reliability and trustworthiness.

**Perturbation Parameters**

- Patch size: randomly chosen within 10%–20% of the total image size.
- Perturbation intensity (ε): varied within the range [2/255, 8/255] in the pixel space.
- Number of adversarial patches: 1–3 patches per image, positioned at random locations.

**Diversity of Attacks**

To maintain robustness, varying patch dimensions, locations, and magnitudes were employed throughout the generation of adversarial attacks, preventing the model from becoming specialized to any single adversarial setup.

**Training Strategy**:

- We created a single adversarial modification for every genuine/manipulated sample.
- The resulting training dataset thus incorporated both original and adversarially altered images.
- The SVM classifier underwent training on ViT-derived features, allowing it to develop decision boundaries that remain stable against adversarial manipulations.

##### Design domain dictionary creation

The final output of Phase 1 is the design domain dictionary, which serves as the enhanced training dataset for the classification phase.

**Dictionary Composition:-** The design domain dictionary contains:

- Original authentic and forged images from the base dataset.
- Augmented versions of all original images (6x increase in size).
- Adversarial examples generated through Patch-Fool attacks.

#### Phase 2: hybrid framework architecture

**Fig. 4:**
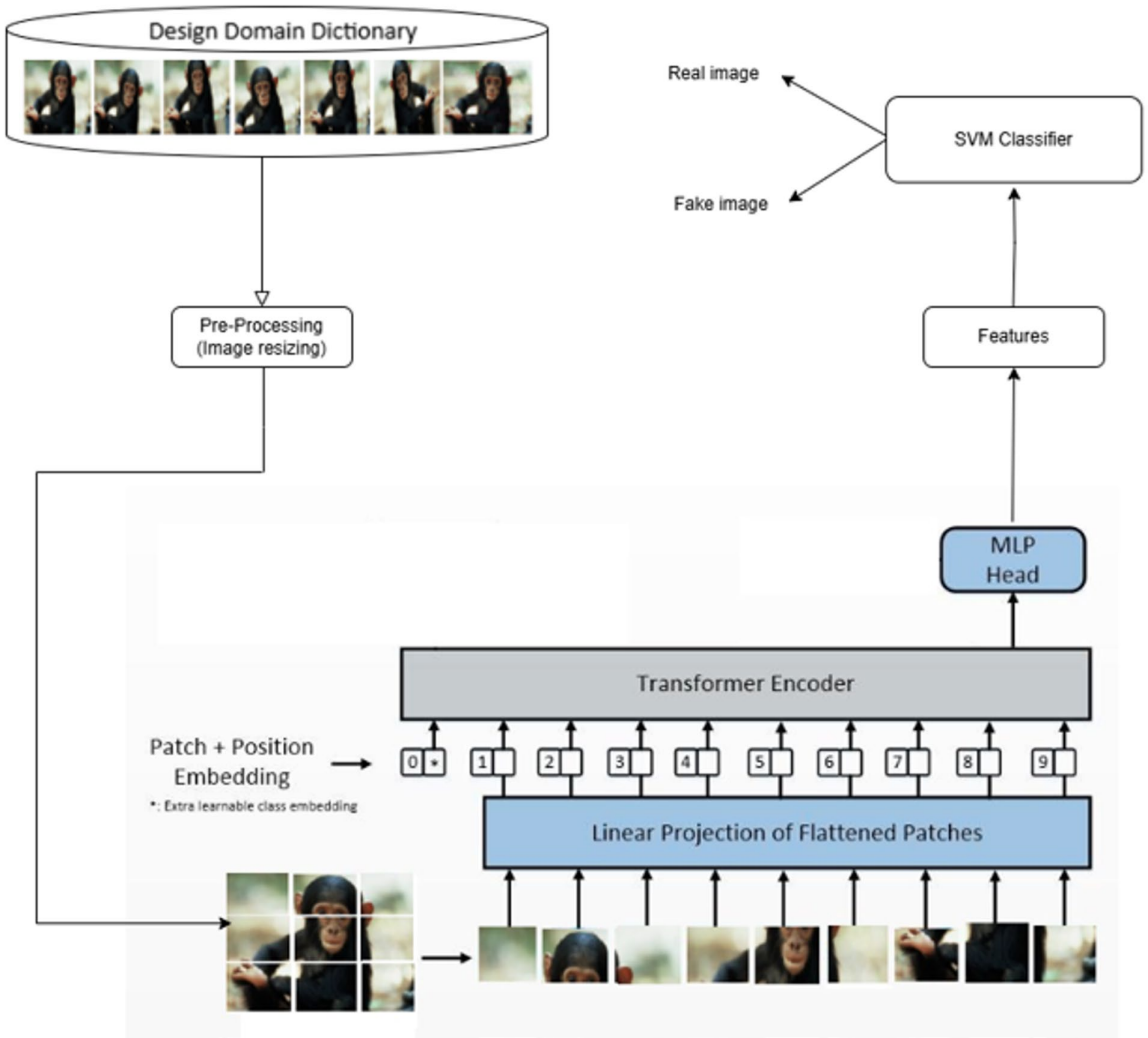
Hybrid framework: images from the design domain dictionary are pre-processed, and their features are extracted by the Vision Transformer and then fed into the SVM for training to distinguish between real and fake images.

The subsequent stage executes the primary classification mechanism using a strategically developed dual-architecture system integrating feature extraction abilities of Vision Transformers alongside classification effectiveness of Support Vector Machines, as demonstrated in Fig. 4. This stage utilizes the design domain repository generated during Phase 1 for developing a resilient image forgery detection framework.

##### Image standardization

The pre-processing step ensures uniformity across all input images:

- **Image Resizing**: All images from the design domain dictionary are resized to 224 × 224 pixels, which is the standard input size for the ViT-B-16 model.
- **Color Space Consistency**: All images are converted to RGB format to maintain color channel uniformity.

##### Feature extraction using vision transformer

**Vision Transformer Architecture:** Selection The feature extraction component utilizes the pre-trained ViT-B-16 model.

**Model Specifications**:

- Architecture: ViT-B-16 with patch size 16 × 16.
- Developer: Google Research.
- Pre-training Dataset: ImageNet-1k.
- Patch Configuration: 196 patches per image (14 × 14 grid).
- Embedding Dimension: 768.
- Transformer Layers: 12.
- Attention Heads: 12 (multi-head self-attention).
- Total Parameters: 86 million.

**Transfer Learning Implementation:** The transfer learning approach is implemented as follows:

- The pre-trained ViT-B-16 model weights are loaded and frozen during feature extraction.
- The final state is extracted as the feature vector.
- Each image is converted into a 768-dimensional feature vector.
- The feature extraction process preserves the rich spatial and semantic information learned from ImageNet.

**Feature Vector Properties:** The extracted feature vectors possess several beneficial characteristics:

- **High Dimensionality**: 768 dimensions capture comprehensive image information.
- **Semantic Richness**: Features encode both low-level and high-level image characteristics.
- **Spatial Awareness**: Transformer architecture maintains spatial relationships between image patches.
- **Transfer Learning Benefits**: Features provide strong baseline representations.

##### Support vector machine training

**Training Data Preparation:** Let X represent the set of extracted feature vectors and y represent the corresponding class labels:

- X = {x₁, x₂, …, xₙ} where x_i_ ∈ ℝ⁷⁶⁸.
- y = {y₁, y₂, …, yₙ} where y_i_ ∈ {0, 1} (0 for authentic, 1 for forged).

**SVM Mathematical Foundation:** Support Vector Machines seek to identify the ideal hyperplane achieving maximum separation between both categories. Given a set of labeled training data points (x₁, y₁), (x₂, y₂), …, (xₙ, yₙ), the SVM^21^ seeks to find a hyperplane defined by:

1 $$\:w\:x+b=0$$

Where:

- w is the weight vector.
- b is the bias term.
- x represents the input feature vector.

**Margin Maximization:** The goal involves maximizing the boundary, representing the distance from the hyperplane to each category’s nearest sample point. The perpendicular measurement from a sample point xi to the hyperplane serves to compute the boundary:

2 $$\text{Margin}\:=\:\frac{1}{\left|\right|w\left|\right|}{|w.x}_{i}+b$$

We use a Support Vector Machine with a Radial Basis Function (RBF) kernel. The regularization parameter was fixed at C = 1.0, and the kernel coefficient γ was set to ‘scale.’

The SVM with RBF kernel was chosen because:

- **High-dimensional embeddings**: The ViT-B/16 produces 768-dimensional feature vectors, and SVMs are well-suited for handling high-dimensional data with limited risk of overfitting.
- **Robust generalization**: SVMs are less sensitive to class imbalance compared to Logistic Regression and showed more stable results in our pilot experiments.
- **Efficiency**: Training an SVM on frozen ViT features is computationally lightweight compared to MLPs.

##### Classification pipeline

The trained hybrid framework processes new, unseen images through a systematic classification pipeline that ensures consistent and reliable results.

**Input Processing:** For each new test image, the system performs:

- Standardization: The input photograph undergoes resizing to 224 × 224 pixels.
- Preprocessing Consistency: The same preprocessing steps applied during training are replicated.
- Format Verification: Ensures the image is in the correct format (RGB, proper dimensions).

**Feature Extraction Process:** In our framework, the Vision Transformer (ViT) functions as a pre-trained feature extraction component. Input images undergo division into non-overlapping patches that receive linear embedding before processing through multiple self-attention mechanisms. The generated embeddings capture a comprehensive feature hierarchy essential for manipulation detection. Lower-level representations identify pixel-based texture anomalies and edge discontinuities commonly found in altered regions. Intermediate embeddings characterize structural and spatial relationships, facilitating identification of copied or inserted content. Higher-level layers encode semantic consistency through modeling extensive spatial dependencies across the complete image, thereby exposing contextual inconsistencies between genuine and modified areas. This hierarchical feature representation proves particularly effective for differentiating original content from manipulated content.

The feature extraction follows the next procedures:

- The preprocessed image is fed into the pre-trained ViT-B-16 model.
- The transformer processes the image through its 12 layers of self-attention.
- The final hidden state is extracted as a 768-dimensional feature vector.
- No additional fine-tuning is performed to maintain consistency with training.

**Classification Decision:** The extracted feature vector is then processed by the trained SVM classifier:

- The feature vector is input to the trained SVM model.
- The SVM applies the learned decision boundary to classify the image.
- The output is a binary decision: 0 (authentic) or 1 (forged).

##### Motivation to hybrid ViT–SVM

###### Limited training data:

- Complete end-to-end fine-tuning of ViTs generally demands extensive, domain-specific datasets. However, image forgery detection datasets are small and diverse in nature. Employing ViT exclusively as a feature extraction mechanism capitalizes on its pre-existing knowledge acquired from ImageNet while preventing overfitting issues.

**SVM Strengths in High-Dimensional Spaces:** The feature representations produced by ViT are characterized by high dimensionality (768-D per image). SVM demonstrates exceptional capability in processing such high-dimensional feature vectors, establishing optimal decision boundaries with robust generalization capabilities despite limited training samples.

**Efficiency :** In comparison to a completely fine-tuned end-to-end ViT, the hybrid model offers superior computational efficiency, requiring training only for the SVM.

### Implementation environment

The trained using a server that uses the operating system Ubuntu 19.01 using Python, Keras, and Tensorflow. The server specification is intel Xeon Processor E5-2640 v2 @ 2.00 GHz ×14, and 60 GB of RAM.

the tests are done on a local machine with the following specifications:

- CPU: 13th Gen Intel(R) Core(TM) i7-13700 (2.10 GHz).
- RAM: 32.0 GB (31.7 GB usable).
- Graphics: Nvidia GeForce GTX 950 M.
- Hard Drive: 1 TB Samsung Evo 860 SSD.
- Operating System: Windows 11 64-bit.

## Results

We will discuss the datasets used in the experiment and the performance metrics that we used to evaluate the proposed method, and we will show the results of the proposed method on the datasets used.

### Dataset

We focused specifically on two classical and widely used manipulation types: splicing and copy-move. These manipulations are directly represented in the benchmark datasets used (CASIA v1.0, CASIA v2.0, MICC-F220, MICC-F2000, and MICC-F600). Other manipulation forms such as AI-generated content were beyond the scope of this work and are considered as directions for future research. We present an extensive compilation of standard datasets employing the copy-move method and the splicing method. The primary features of individual datasets appear summarized within Table 1.

**Table 1:** Copy-move/splicing datasets.

Dataset	Manipulation	Authentic/ Forged	Format
CASIA1	copy-move, splicing	750/975	JPG
CASIA2	copy-move, splicing	7491/5123	JPG, BMP, TIF
MICC-F220	copy-move	110/110	JPG
MICC-F600	copy-move	440/160	JPG, PNG
MICC-F2000	copy-move	1300/700	JPG

**CASIA v1**^22^: It includes 1725 color JPEG photos with a pixel resolution of 384 × 256. 975 of these photos are fake, but the others are authentic. It includes both splicing and copy-move attacks.

**CASIA v2**^22^: It includes 5123 forged color photos at various sizes in addition to 7491 real ones. The picture formats include TIFF, BMP, and JPEG. It includes splicing and copy-move attacks.

**MICC-F220**^23^: This comprises 110 copy-moved photographs alongside 110 authentic JPEG color photographs. The duplicated segments additionally undergo various post-processing operations, encompassing noise incorporation, resizing, and rotation.

**MICC-F600**^23^: This includes 440 authentic photographs and 160 altered color photographs within JPEG and PNG representations.

**MICC-F2000**^23^: This encompasses 700 copy-moved and 1300 authentic JPEG photographs, individually sized at 2048 × 1536 pixels.

### Performance metrics

Precision metric assesses the framework’s correctness when detecting positive cases. The measure calculates true positive identifications relative to the combination of true positives and false positives.

3 $$\:Percision=\:\frac{TP}{TP+FP}$$

Recall measures the framework’s ability to identify all positive examples. This value is derived from dividing true positive predictions by the combined count of false negatives and true positives.

4 $$\:\:\:\:\:\:\:\:\:\:\:\:\:\:\:\:\:\:Recall=\:\frac{TP}{TP+FN}$$

F1-score constitutes a holistic indicator of framework performance calculated using the harmonic mean between precision and recall. The metric ranges between 0 and 1.

5 $$\:\:\:\:\:\:\:\:\:\:\:\:\:\:\:\:\:F1-score=2\left(\frac{Precision\:.\:\:Recall}{Precision+Recall}\right)\:\:$$

Accuracy quantifies how accurate the model’s predictions are overall.

6 $$\:\:\:\:\:\:\:\:\:\:\:\:\:\:\:\:Accuracy=\:\frac{TP+TN}{TP+FP+FN+TN}$$

### Results

Within this section, initially, we examine the pre-processing dataset stage outcomes. Subsequently, We analyze the experimental findings obtained through applying the introduced approach. Finally, we evaluate the introduced approach against current techniques addressing the identical challenge.

#### Processing the dataset phase results

During the dataset preparation stage for individual images within CASIA v1, CASIA v2, MICC-F220, MICC-F600, and MICC-F2000 repositories, we produce multiple images through data augmentation methods, plus one adversarial sample, as illustrated in Fig. 3. Consequently, we obtain the design domain repository for the respective datasets. Subsequently, the design domain repository serves to train the introduced framework.

For all datasets, including CASIA v1.0, CASIA v2.0, MICC-F220, MICC-F600, MICC-F2000, and the merged dataset, we employed a stratified 80/20 split with balanced classes. 80% of the data were used for training, while the remaining 20% were reserved exclusively for testing. To strictly prevent data leakage, the splitting of the merged dataset was performed after merging all individual datasets. In addition, we conducted a 5-fold cross-validation, where in each fold, 80% of the data were used for training and 20% for testing.

#### The learning and classification phase results

We evaluated the introduced method’s effectiveness for image forgery detection using six separate repositories: CASIA v1.0, CASIA v2.0, MICC-F220, MICC-F2000, MICC-F600, and the Merged Dataset, where we integrated all aforementioned datasets. Precision, recall, F1 measure, and accuracy underwent calculation for delivering a thorough assessment. In addition to a single stratified 80/20 split, we also performed 5-fold cross-validation to provide statistical reliability. The cross-validation results are reported as mean ± standard deviation (std) across folds, as shown in Tables 2 and 3. These findings confirm that the high performance is stable and not the result of overfitting or a biased data split.

**Fig. 5:**
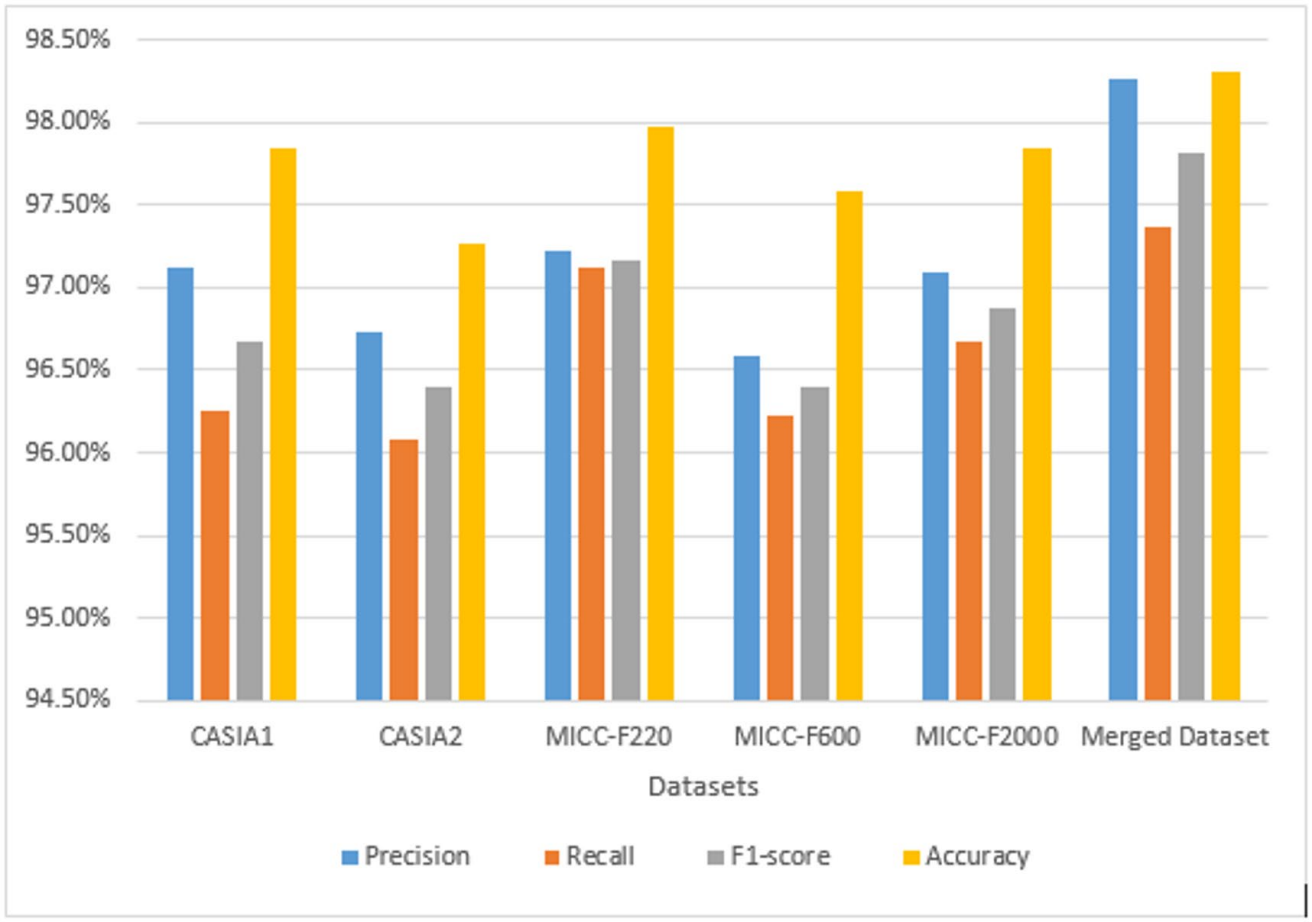
Comparative performance metrics of the proposed method for forgery detection across multiple datasets with no augmentation techniques.

**Fig. 6:**
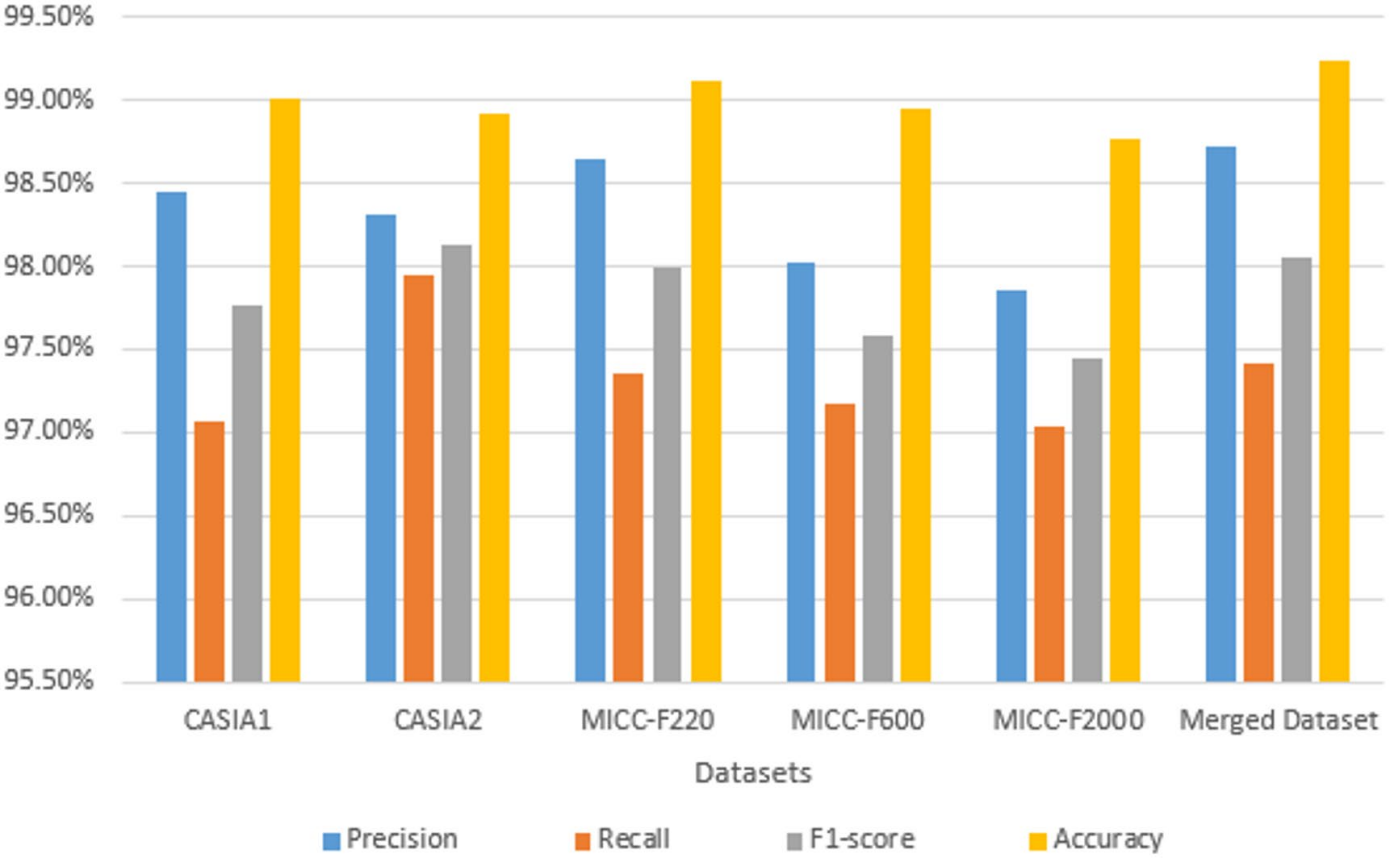
Comparative performance metrics of the proposed method for forgery detection across multiple datasets with augmentation techniques.

We conducted two experiments. During the initial experiment, where we evaluated the introduced framework across the six repositories without implementing data augmentation methods within the dataset processing stage, we implemented solely the adversarial perturbation. The resulting experimental outcomes appear within Table 2; Fig. 5. Specifically, regarding CASIA v1.0, the introduced framework demonstrated accuracy reaching 97.85%±0.61, with precision, recall, and F1-measure scores achieving 97.12%±0.42, 96.25%±0.2, and 96.68%±0.44, respectively. Comparable impressive outcomes appeared throughout CASIA v2.0, MICC-F2000, MICC-F600, MICC-F220, and the Merged Dataset, emphasizing the framework’s adaptability and performance across varied conditions. On the merged dataset, the proposed model achieved an average accuracy of 98.31% ± 0.22 and F1-score of 97.81% ± 0.23, demonstrating consistent results across all folds. During the subsequent experiment, we evaluated the introduced framework using the six repositories by implementing data augmentation methods alongside adversarial perturbation methods within the dataset processing stage. The resulting experimental outcomes appear withinTable 3; Fig. 6. Specifically, regarding CASIA v1.0, the introduced framework demonstrated accuracy reaching 99.01%±0.21, with precision, recall, and F1-measure scores achieving 98.45%±0.32, 97.06%±0.28, and 97.76%±0.25, respectively. Comparable impressive outcomes appeared throughout CASIA v2.0, MICC-F2000, MICC-F600, MICC-F220, and the Merged Dataset. On the merged dataset, the proposed model achieved an average accuracy of 99.23% ± 0.27 and F1-score of 98.06% ± 0.3, demonstrating consistent results across all folds. We additionally discovered that effectiveness improves through implementing data augmentation methods.

Beyond the Patch-Fool adversarial samples incorporated during the training phase, we performed additional analyses using alternative adversarial attack methodologies, specifically Patch-Fool, FGSM and PGD. For FGSM testing, we employed perturbation intensity levels of ε = 8/255, whereas PGD evaluation utilized ε = 8/255 with 10 iterations and a step increment of ε/10. The outcomes, presented in Table 4, demonstrate that our framework maintains strong resilience against Patch-Fool attacks (achieving 98.2% accuracy on CASIA v1.0 and 95.4% on the combined dataset), whereas performance shows greater deterioration when subjected to FGSM and particularly PGD attacks, which aligns with expectations. However, the AUC score of 0.88 under PGD conditions indicates that the model retains a substantial level of discriminative capability. These results validate the robustness of our methodology while simultaneously identifying areas requiring additional research to enhance defense mechanisms against more different adversarial attacks.

We also compute the average run time of the proposed method across all the datasets. As shown in Table 5, the average run time of the proposed method on the CASIA1 dataset is (221.74 ms) when testing 345 image and on the Merged Dataset is (341.82 ms) when testing 3431 image.

**Table 2:** The performance of the proposed method with no augmentation (5-fold cross-validation results (.

Dataset	Precision (mean ± std)	Recall (mean ± std)	F1-score (mean ± std)	Accuracy (mean ± std)
CASIA1	97.12 ± 0.42	96.25 ± 0.2	96.68 ± 0.44	97.85 ± 0.61
CASIA2	96.73 ± 0.80	96.08 ± 0.71	96.4 ± 0.34	97.27 ± 0.27
MICC-F220	97.22 ± 0.41	97.12 ± 0.3	97.16 ± 0.36	97.98 ± 0.34
MICC-F600	96.59 ± 0.52	96.23 ± 0.51	96.4 ± 0.52	97.58 ± 0.54
MICC-F2000	97.09 ± 0.30	96.68 ± 0.20	96.88 ± 0.43	97.85 ± 0.47
Merged Dataset	98.27 ± 0.24	97.37 ± 0.02	97.81 ± 0.23	98.31 ± 0.22

**Table 3:** The performance of the proposed method with augmentation (5-fold cross-validation results (.

Dataset	Precision (mean ± std)	Recall (mean ± std)	F1-score (mean ± std)	Accuracy (mean ± std)
CASIA1	98.45 ± 0.32	97.06 ± 0.28	97.76 ± 0.25	99.01 ± 0.21
CASIA2	98.31 ± 0.41	97.95 ± 0.36	98.13 ± 0.33	98.92 ± 0.29
MICC-F220	98.64 ± 0.38	97.35 ± 0.35	97.99 ± 0.31	99.12 ± 0.26
MICC-F600	98.02 ± 0.29	97.18 ± 0.27	97.59 ± 0.24	98.95 ± 0.22
MICC-F2000	97.85 ± 0.34	97.03 ± 0.30	97.44 ± 0.28	98.76 ± 0.25
Merged Dataset	98.72 ± 0.36	97.41 ± 0.33	98.06 ± 0.30	99.23 ± 0.27

**Table 4:** Comparison proposed method under different adversarial attacks.

Attack (parameters)	CASIA v1.0 Accuracy	CASIA v1.0 AUC	Merged Accuracy	Merged AUC
Patch-Fool (32 × 32, ε = 8/255)	98.20%	0.986	95.40%	0.970
FGSM (ε = 8/255)	95.0%	0.960	91.5%	0.948
PGD (ε = 8/255, 10 steps)	88.5%	0.905	84.0%	0.880

**Table 5:** The average run time of the proposed method datasets.

Dataset	Number of images	Run Time (ms)
CASIA1	345	221.74
CASIA2	2522	312.56
MICC-F220	44	254.78
MICC-F600	116	291.34
MICC-F2000	400	322.65
Merged Dataset	3431	341.82

#### Result comparison

Our introduced framework surpassed current techniques in image manipulation detection. Table 6 presents the outcomes of image manipulation detection using the introduced framework versus existing models. When compared against existing methods (CNN, Shallow NN, EBSA–SVM), as well as the end-to-end ViT baseline, our hybrid ViT + SVM consistently delivered superior results. For instance, on MICC-F220, it achieved 99.12% ± 0.26 accuracy with AUC = 0.9745, surpassing EBSA + LS-SVM (98.6%). On the merged dataset, the proposed framework achieved 99.23% ± 0.27 accuracy with AUC = 0.9854, which is the best among all methods tested. These comparisons highlight that integrating transformer-based feature extraction with SVM classification improves both accuracy and robustness, indicating strong potential for generalization to diverse datasets.

**Table 6:** Comparison of existing models with the proposed Method.

Dataset	Technique	Accuracy (mean ± std)	AUC
CASIA1	CCA classifier^19^	98.79%	-
	ViT^31^	98%	-
	**ViT + SVM (ours)**	**99.01%± 0.21**	**0.9823**
CASIA2	CNN^24^	92.3%	-
	Shallow Neural Network^25^	97.94%	-
	ViT^31^	98%	-
	**ViT + SVM(ours)**	**98.92%± 0.29**	**0.9875**
MICC-F220	(CNN) fine-tuned with (SCFF)^26^	97.64%	-
	EBSA + LS-SVM^27^	98.6%	-
	**ViT + SVM(ours)**	**99.12%±0.26**	.**9745**
MICC-F600	EBSA + LS-SVM^27^	98.3%	-
	ViT^31^	98%	-
	**ViT + SVM(ours)**	**98.95%± 0.22**	**0.9761**
MICC-F2000	EBSA + LS-SVM^27^	98.6%	-
	CNN^28^	98.52%	-
	ViT^31^	96%	-
	**ViT + SVM(ours)**	**98.76%± 0.25**	**0.9632**
Merged Dataset	**ViT + SVM(ours)**	**99.23% ± 0.27**	**0.9854**

### Attention map visualizations under adversarial attacks

We investigate the robustness of our proposed image forgery detection framework. We visualize the attention maps of the Vision Transformer (ViT) feature extractor under both clean and adversarial inputs. Our proposed method employs a pre-trained ViT as a feature extractor and an SVM as the classifier. While the SVM itself operates only on the extracted features, the internal attention patterns of the ViT provide interpretability into how adversarial perturbations influence feature quality.

**Fig. 7:**
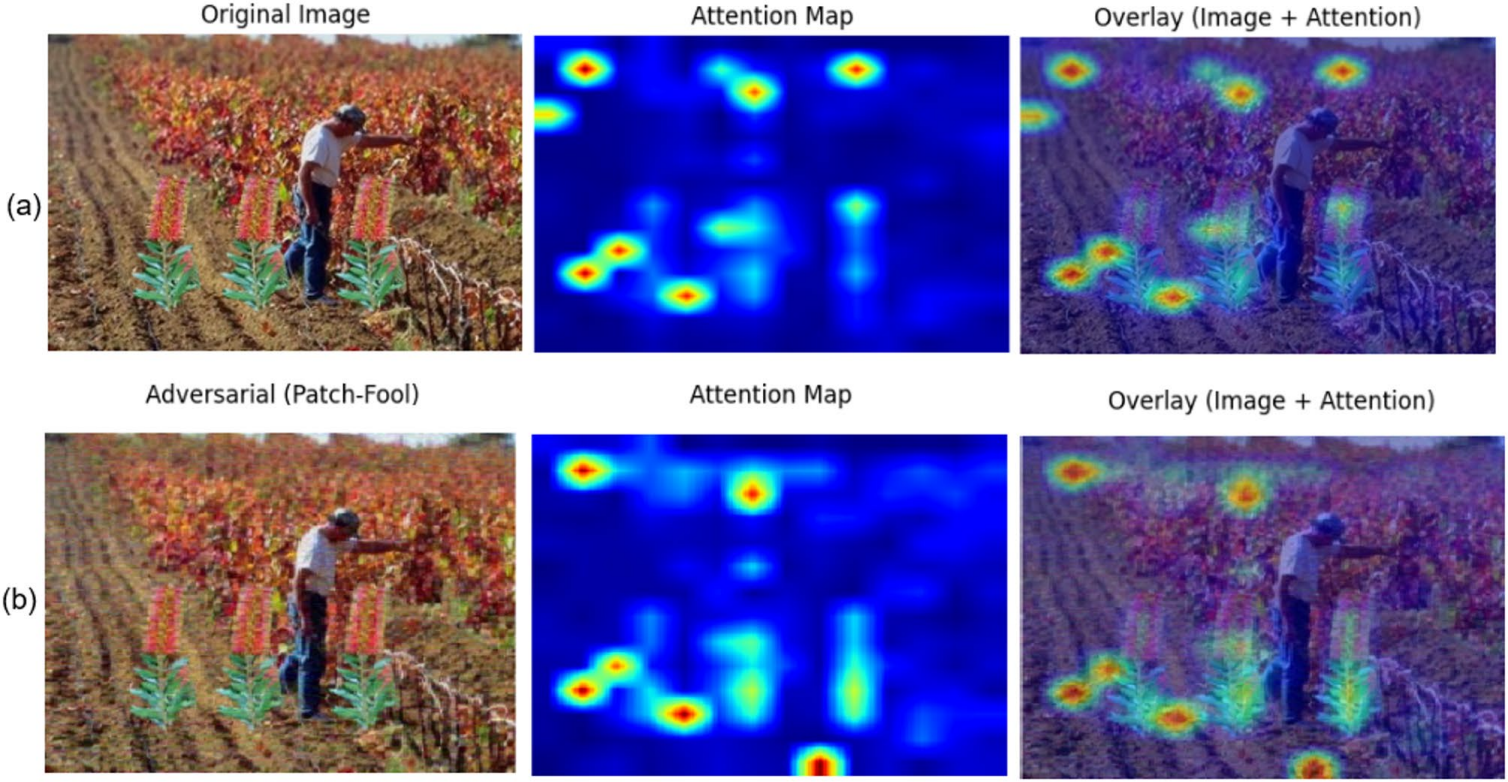
Visualization of attention maps for **(a)** a clean input image and **(b)** its adversarial counterpart. In **(a)**, the Vision Transformer attends to semantically relevant regions that are critical for detecting image forgery. In **(b)**, the adversarial perturbation (Patch-Fool) redirects the model’s attention to irrelevant areas, which degrades the feature representation and can mislead the downstream SVM classifier.

Figure 7.a shows the attention maps of a clean image. The ViT focuses on semantically relevant regions that are important for detecting forgeries. In contrast, Fig. 7.b shows the same example after applying an adversarial perturbation (Patch-Fool attack). Here, the attention is redirected toward irrelevant background regions, which disrupts the representation and can mislead the downstream SVM. These results demonstrate that adversarial attacks not only alter classification outcomes but also distort the internal feature extraction process.

To mitigate this vulnerability, we adopted adversarial training. Specifically, adversarial examples were injected during the learning process, allowing the model to learn more robust representations. By exposing the ViT–SVM pipeline to perturbed inputs, adversarial training encourages the feature extractor to maintain discriminative attention patterns even under attack, thereby improving robustness in image forgery detection.

### Failure case analysis

**Fig. 8:**
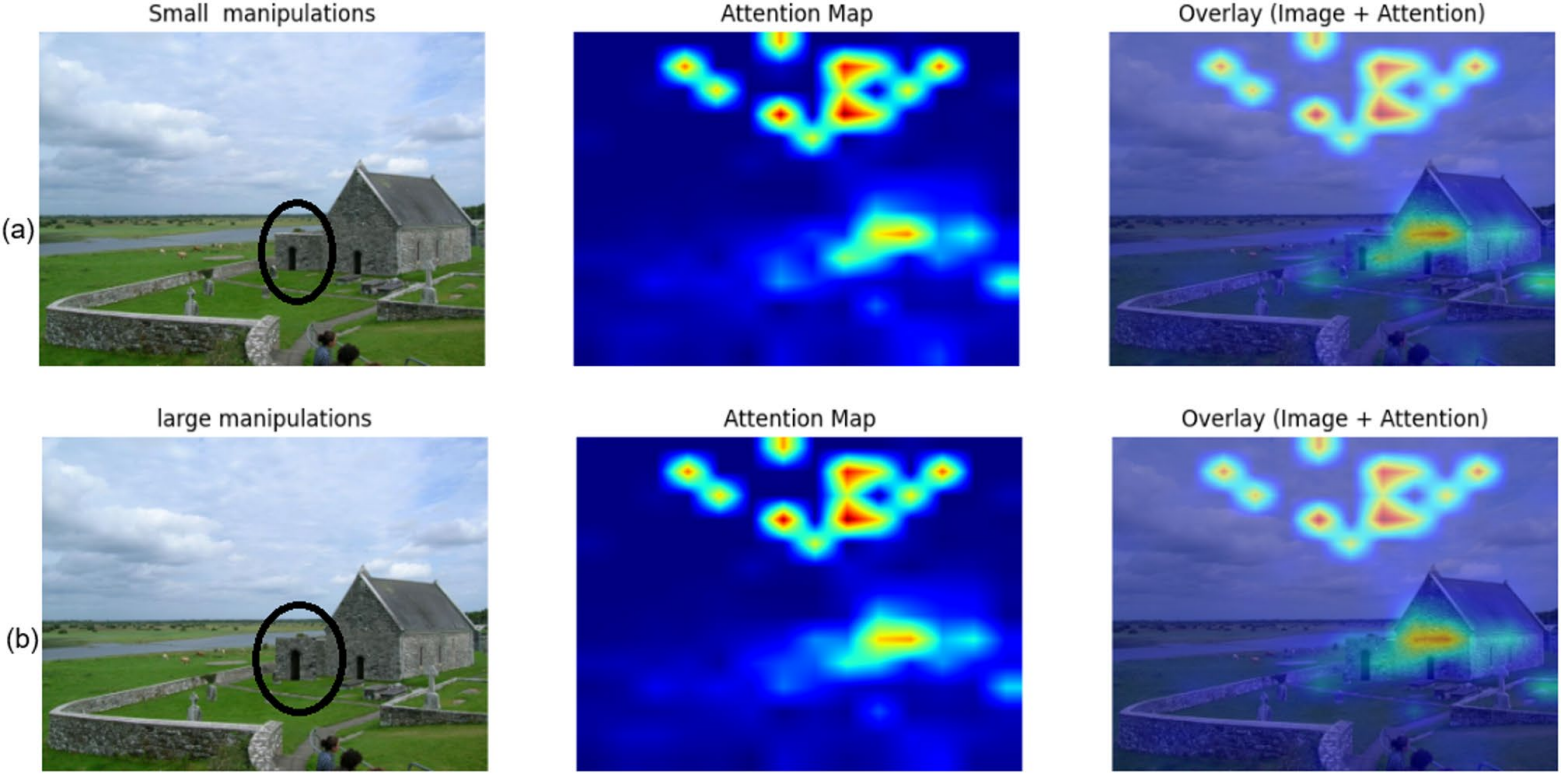
Attention map analysis of failure and success cases. **(a)** Small manipulation that was misclassified due to subtle pixel-level changes not captured by the model’s attention. **(b)** Large manipulation was correctly detected, where strong texture and structural inconsistencies attracted the attention mechanism.

Athough the hybrid ViT–SVM framework achieves robust detection in most cases, we observed failure cases where the system misclassified images with very small manipulations. Figure 8.a demonstrates such a scenario, where only a subtle modification was introduced. The attention map indicates that the model primarily focused on the overall scene context and ignored the manipulated region, leading to a false negative classification.

In contrast, Fig. 8.b shows an example of large manipulations, where the tampered area produced significant texture and structural inconsistencies. These discrepancies were successfully highlighted in the attention map and correctly classified as manipulated.

This comparative analysis reveals that the proposed system is more effective at detecting large-scale or perceptually significant forgeries, while fine-grained manipulations remain challenging. Addressing this limitation represents an important avenue for future improvement.

## Discussion

This work specifically targets copy-move and splicing forgeries, as they are the most widely studied and critical manipulation types in current forensic benchmarks. Future work will focus on extending the framework to modern AI-driven forgeries, including deepfake and GAN-generated content, to address the evolving landscape of digital image manipulation.

The experimental results demonstrated consistently strong performance across six standard forensic datasets (CASIA v1.0, CASIA v2.0, MICC-F220, MICC-F600, MICC-F2000, and the merged dataset). Within both experimental configurations—utilizing solely adversarial training or integrating it alongside data enhancement—the approach regularly obtained elevated accuracy, precision, recall, and F1-measures. These results validate that the dual architecture, combining Vision Transformer (ViT) alongside an SVM algorithm, demonstrates substantial capability for differentiating between genuine and altered photographs.

### Impact of data augmentation and adversarial training

A key observation is the significant improvement in performance when both data augmentation and adversarial training were employed in the pre-processing phase. Data augmentation increased the diversity of training samples by simulating real-world transformations such as rotation, translation, and scaling, while adversarial training improved robustness against malicious perturbations. Together, these strategies expanded the design domain dictionary and allowed the model to generalize better across multiple datasets. The results highlight that augmentation techniques are sufficient in the context of forgery detection and that resilience against adversarial attacks is crucial for practical deployment.

### Impact of transfer learning

A nother important aspect of the proposed framework is the use of transfer learning through a pre-trained Vision Transformer (ViT-B/16) model. Instead of training from scratch, which requires very large-scale datasets and extensive computational resources, transfer learning leverages knowledgeacquired from ImageNet-1k and adapts it to the forgery detection task. This approach had two main merits: (i) better generalization across different datasets, and (ii) quicker convergence during training. The results demonstrate that transfer learning has been a key factor that helps the hybrid ViT-SVM framework to outperform the traditional CNN-based models and achieve state-of-the-art results on all six benchmark datasets.

### Impact of hybrid framework

**Robust feature extraction:**

- ViT model, leveraging pre-training on extensive datasets such as ImageNet, generates comprehensive and transferable feature representations that encompass both localized patch-level details and broader contextual relationships.
- These features are highly discriminative and generalizable.

**Efficient & Interpretable Classification:**

- Rather than incorporating additional fully connected layers for end-to-end classification, we utilize SVM, which excels in high-dimensional feature spaces and is effective in handling small-to-medium training datasets while avoiding overfitting issues.
- SVM establishes a well-defined decision boundary through the maximum-margin principle, exhibiting reduced susceptibility to overfitting compared to deep classification layers when working with limited dataset sizes.

**Empirical Observations:**

- Our comparative analysis reveals that the hybrid approach surpasses standalone end-to-end ViT fine-tuning in both classification accuracy and resilience to adversarial manipulations.
- For instance, whereas end-to-end fine-tuned ViT obtained 98% accuracy on CASIA v1.0, our hybrid ViT + SVM configuration achieved 99.01%± 0.21 accuracy.

### Comparison with existing methods

In comparison to the state-of-the-art forgery detection methods, the proposed method consistently achieved competitive or superior performance across all datasets. This main advantage can be attributed to the ViT’s capacity to learn global dependencies of images through its attention mechanism, and the SVM’s powerful discriminative capacity in splitting high-dimensional score spaces. Unlike CNN-based models, which are very vulnerable to subtle perturbations, the hybrid ViT-SVM model was very robust to adversarial manipulations while maintaining high accuracy.

### Strengths and limitations

The strength of the proposed framework lies in its dual strategy of dataset enrichment and hybrid classification. By incorporating adversarial examples during training, the model not only demonstrated enhanced robustness but also showed improved adaptability across different datasets. This highlights the potential of integrating transformer-based representations with SVM decision boundaries for reliable image forgery detection.

Nevertheless, several limitations should be acknowledged.

First, the generation and integration of adversarial examples (e.g., Patch-Fool attacks) introduce additional computational overhead during dataset preparation and training, which may limit scalability for larger datasets or real-time applications.

Second, although the model achieved high accuracy across six benchmark datasets, these datasets represent controlled and curated conditions. Therefore, the model’s generalization to real-world, large-scale social media images—where variations in compression, lighting, and contextual manipulations occur—still requires further validation.

Third, the current framework addresses binary classification (real vs. forged), which simplifies the problem but does not fully capture the complexity of multi-class forensic scenarios such as distinguishing splicing, copy-move, and AI-generated forgeries. Addressing this limitation would enhance the system’s forensic interpretability and robustness.

Finally, the study focuses primarily on static images, leaving temporal or multimodal extensions such as video for future exploration.

Overall, while these limitations outline the boundaries of the current work, they also provide clear directions for future research aimed at improving scalability, generalization, and applicability in complex real-world forensic settings.

### Practical implications

The findings suggest that the proposed approach can be effectively integrated into multimedia forensics systems, social media monitoring platforms, and digital evidence verification pipelines. The proposed method demonstrated high resilience against adversarial perturbations, suggesting its potential applicability in high-stakes domains such as cybersecurity and digital forensics, where reliability is crucial.

### Adaptability to emerging manipulation techniques

A key advantage of the developed ViT-SVM hybrid architecture stems from its modular structure. The Vision Transformer component, pre-trained on extensive natural image collections (ImageNet-21k), produces high-level semantic representations that transcend specific manipulation categories. These representations encompass both global relationships (image composition, object coherence) and localized anomalies (surface patterns, boundaries), ensuring adaptability to emerging manipulation techniques.

Furthermore, the SVM-based classification component enables computationally efficient adaptation. Accommodating new forgery categories (e.g., AI-generated manipulations including diffusion-based inpainting or GAN-generated face replacements) necessitates only SVM retraining using ViT-derived feature vectors, eliminating the requirement for costly comprehensive model retraining.

Although we restrict our evaluation to static images, the proposed ViT-SVM framework could be adapted for videos by applying frame-level feature extraction and modeling temporal consistency across consecutive frames. This direction is highlighted as a potential avenue for future research.

For large-scale deployment (e.g., on social media), Vision transformer (ViT) models are indeed computationally intensive, we addressed this challenge in several ways:

- Feature extraction only – In our framework, the pre-trained ViT is used solely for feature extraction rather than full end-to-end fine-tuning, which reduces computational cost.
- Lightweight classification – The extracted features are classified using an SVM, which is computationally efficient and suitable for real-time inference.
- Batch processing – The system can be parallelized to process multiple images simultaneously, leveraging GPU acceleration available on most cloud platforms.

## Conclusion and future work

The dissemination of doctored multimedia via social media is a serious threat to information integrity, security and trust. The recent Vision Transformer model (ViT) can serve as an alternative to Convolutional Neural Networks (CNNs). Vision Transformers (ViTs) are unique in their architecture and in focusing their self-attention to learn complex features in an effective way.

In this paper, we propose utilizing a hybrid deep learning model combining a pretrained Vision Transformer (ViT) for feature extraction and a Support Vector Machine (SVM) for binary classification to distinguish real versus fake images. To further improve the robustness of our proposed Method, we further employ adversarial training to make the model robust to adversarial attacks, and apply data augmentation techniques to augment the dataset size. Results from experiments on several benchmark datasets, including CASIA v1.0, CASIA v2.0, MICC-F2000, and MICC-F600, demonstrate that our proposed method performs effectively in identifying image forgeries. The proposed method offers a solution for detecting manipulated images, which will help social media platforms, cybersecurity systems, and forensic investigations in tackling the growing issue of manipulated media.

Although the developed ViT–SVM framework achieved high accuracy and resilience against adversarial manipulations across various benchmark datasets, several constraints warrant recognition. Initially, the assessment focused exclusively on conventional manipulation categories (splicing and copy-move), while alternative forms, including AI-generated synthetic content and large-scale real-world datasets remain unexamined. Second, while the present work focuses on image-level forgery detection, the motivation extends naturally to video forgeries. Third, the adversarial resilience was predominantly evaluated against Patch-Fool attacks; despite supplementary experiments with FGSM and PGD yielding encouraging outcomes, more sophisticated and adaptive attack strategies require further investigation to fully assess the framework’s generalization and defense capabilities. Moreover, computational overhead introduced during adversarial training may affectscalability in large-scale scenarios.

For subsequent research directions, we intend to (i) expand the framework to accommodate a broader range of manipulation methodologies, including GAN-based forgeries and AI-generated images like deepfakes, (ii) investigate advanced defensive mechanisms against sophisticated adversarial attacks, (iii) future work could explore extending this approach to video forgery detection, improving generalization across diverse manipulation techniques, and AI-generated content. By advancing forgery detection methods, we contribute to mitigating the harmful societal impacts of fake multimedia content.

## Data Availability

The datasets that support the findings of this research are available in the following: CASIA1:- https://www.kaggle.com/datasets/sophatvathana/casia-dataset.CASIA2:-https://www.kaggle.com/datasets/divg07/casia-20-image-tampering-detection-dataset.MICC-F220:-https://www.kaggle.com/datasets/mashraffarouk/micc-f220.MICC-F600:- https://www.kaggle.com/datasets/nishaahin/miccf600.MICC-F2000:-https://www.kaggle.com/datasets/manas29/micc-f2000.
